# Exercise Exerts Antidepressant Effects via Endoplasmic Reticulum Stress Modulation: Molecular Mechanisms and Research Progress

**DOI:** 10.3390/biology15110836

**Published:** 2026-05-27

**Authors:** Xin-Yue Zhou, Yu-Wei Liu, Cheng-Hao Zhong, Ran Xu, Kang Guan, Hao-Nan Li, Jia-Ting Huang, Ke Xue, Yi Wang, Xiang-He Chen

**Affiliations:** College of Physical Education, Yangzhou University, Yangzhou 225127, China; zhouxinyuegou@163.com (X.-Y.Z.); yangdayw@163.com (Y.-W.L.); 18950056613@163.com (C.-H.Z.); xuran2002@yeah.net (R.X.); 18236178987@163.com (K.G.); lisi_9527@163.com (H.-N.L.); huangjiating8012@163.com (J.-T.H.); 13775633406@163.com (K.X.); 18919232176@163.com (Y.W.)

**Keywords:** depression, endoplasmic reticulum stress, movement, molecular mechanism

## Abstract

Abnormal activation of endoplasmic reticulum stress (ERS) is an important driving factor for the occurrence and development of depression. As a safe and effective non-drug intervention, exercise plays an important role in improving depression-like behavior. This paper systematically reviews the core role of ERS-mediated neuronal apoptosis, inflammatory response, calcium homeostasis imbalance and autophagy abnormalities in depression and focuses on the molecular mechanism of exercise to improve depression by regulating ERS and its downstream signals. Existing evidence shows that exercise can exert antidepressant effects through multi-level remodeling of ERS-related signaling networks, including inhibiting UPR-related apoptosis, inhibiting microglial pro-inflammatory polarization, regulating MAMs-mediated calcium-mitochondrial interaction, and restoring autophagy activity (LC3-II, Beclin-1). Although the above findings provide a new potential perspective for explaining the antidepressant effect of exercise, its precise intervention application is still limited by the lack of population research, unclear selection of exercise types and large differences in exercise intensity. This article aims to review and analyze the mechanism of endoplasmic reticulum stress in the improvement of depression by exercise, in order to provide a new theoretical reference for the prevention and treatment of depression.

## Simple Summary

Depression is a common mental health disorder that can damage brain function and reduce quality of life. Recent studies suggest that cellular stress affecting the endoplasmic reticulum, a structure responsible for maintaining normal cell function, may contribute to depression by promoting inflammation, nerve cell damage, and impaired brain communication. Exercise is widely recognized as a safe and effective non-drug treatment for depression, but its biological mechanisms are not fully understood. This review summarizes current evidence showing that exercise may improve depressive symptoms by reducing harmful cellular stress, protecting brain cells, maintaining calcium balance, improving mitochondrial function, and enhancing the removal of damaged cellular components. These effects may help restore normal brain activity and emotional regulation. Although most findings are based on animal studies and further clinical research is needed, current evidence supports exercise as a promising supportive strategy for the prevention and treatment of depression.

## 1. Introduction

Depression is a highly prevalent mental health disorder. The World Health Organization has recognized depression as one of the leading causes of disability worldwide [1]. It markedly impairs physical and mental health as well as social functioning, increases mortality risk, and has become a global public health problem requiring urgent attention [2].

Recent evidence indicates that dysfunction of intracellular organelles, including the endoplasmic reticulum (ER), lysosomes, and mitochondria, together with disturbed inter-organelle communication, plays an important role in the pathophysiology of depression. As a central membranous organelle in eukaryotic cells, the ER is highly responsive to alterations in the cellular microenvironment. When cells are exposed to stimuli such as ischemia, hypoxia, acidosis, oxidative stress, or calcium homeostasis imbalance, protein folding is disrupted, leading to the accumulation of unfolded and misfolded proteins in the ER and thereby disturbing ER homeostasis and function [3]. Under such conditions, cells initiate a self-protective response known as endoplasmic reticulum stress (ERS) [4]. Excessive ERS can lead to apoptosis, inflammatory responses, Ca^2+^ homeostasis imbalance, oxidative stress, and autophagy inhibition, thereby contributing to the onset and progression of major depressive disorder (MDD) [5]. Analyses of depression-related brain regions, including the hippocampus, prefrontal cortex, amygdala, and striatum, in chronic restraint stress (CRS) animal models have shown significantly increased expression of ERS-related proteins, such as GRP78, ATF6, XBP1, and CHOP, suggesting that ERS may be an important mechanism regulating depression. In addition, persistent activation of the ERS system has been reported in patients with depression [4,6]. However, although multiple studies have implicated ERS in depression, direct causal evidence linking ERS to core pathological manifestations remains limited. Moreover, the integrated interactions among ERS, inflammation, hypothalamic–pituitary–adrenal axis dysfunction, mitochondrial abnormalities, and impaired autophagy have yet to be fully elucidated, and the translational value of targeting these pathways requires further confirmation.

Exercise, as a healthy lifestyle strategy, has been widely applied in the management of neurological and cognitive disorders such as Alzheimer’s disease (AD), Parkinson’s disease (PD), depression, and anxiety disorders [7,8]. In recent years, exercise has shown broad application prospects in the field of depression because of its multiple biological effects, including anti-inflammatory and antioxidant actions, regulation of monoamine neurotransmitter synthesis and release, and promotion of neuroplasticity [9,10,11]. Meanwhile, a recent animal study found that six weeks of treadmill exercise improved CUMS-induced depression-like behaviours in mice by activating the classical Nrf2/Keap1 antioxidant pathway and reducing ERS [12]. Although this finding highlights the potential of exercise to alleviate depression by targeting ERS, the downstream molecular mechanisms remain incompletely understood, particularly because direct verification of the causal chain of “exercise-ERS regulation-depression improvement” is still lacking. Therefore, to systematically clarify the role of ERS in exercise-related improvement of depressive disorders and provide a theoretical basis for precise exercise interventions, this review summarizes ERS-mediated antidepressant effects of exercise and the potential underlying mechanisms.

## 2. Method

To identify relevant studies investigating the role of endoplasmic reticulum stress (ERS) in exercise-mediated improvement of depressive disorders, a comprehensive literature search was conducted. Electronic databases including PubMed, Web of Science, and CNKI were searched for articles published up to 21 May 2026. The search strategy utilized a combination of the following core terms: “exercise” OR “physical activity”, “endoplasmic reticulum stress” OR “ERS”, and “depression” OR “depressive-like behavior”. These were combined with specific keywords including “unfolded protein response (UPR)”, “PERK”, “IRE1α”, “ATF6”, “Ca^2+^ homeostasis”, “autophagy”, “neuroinflammation”, “mitochondria”, “animal model”, “clinical study”, “mechanism”, and “signaling pathway”. We included both preclinical and clinical studies to provide a comprehensive overview. Exclusion criteria comprised non-English publications (with the exception of Chinese literature from CNKI), conference abstracts, letters, editorials, dissertations, and non-peer-reviewed articles.

## 3. Role of Endoplasmic Reticulum Stress in Depression

### 3.1. Unfolded Protein Response

After ERS occurs, cells maintain proteostasis by activating the UPR; however, when ERS persists or the intensity of stimulation exceeds the compensatory capacity of cells, the UPR may shift from an adaptive response to pro-apoptotic and pro-inflammatory signalling, thereby contributing to the development and progression of depression. The UPR is mainly mediated by three signaling pathways: protein kinase RNA-like endoplasmic reticulum kinase (PERK), inositol-requiring enzyme 1α (IRE1α), and activating transcription factor 6 (ATF6). These pathways influence neuronal functional homeostasis by regulating protein translation, inflammatory responses, oxidative stress, and apoptosis, and are strongly linked to neuronal injury observed in depression and abnormal neuroplasticity [13].

Under persistent ERS, UPR-regulated adaptive mechanisms may fail to meet the sharply increased demand for protein folding, thereby inducing apoptotic programmes [14] and ultimately contributing to depression [15] This process triggers apoptosis through multiple molecular mechanisms, including the IRE1/TRAF2/ASK1/JNK axis, CHOP-mediated transcriptional regulation, and caspase-12-mediated ER-specific apoptosis [16,17]. Specifically, after activation, IRE1α can recruit tumour necrosis factor receptor-associated factor 2 (TRAF2), increase the expression of apoptosis signal-regulating kinase 1 (ASK1), activate the JNK signalling pathway, and induce apoptosis [18]. Under severe or prolonged ERS, PERK, IRE1, and ATF6 jointly promote upregulation of CHOP. CHOP enhances neuronal sensitivity to ERS by downregulating Bcl-2 and upregulating BIM, thereby aggravating depression-related neural injury [19]. In addition, ERS specifically activates caspase-12, initiates the caspase cascade, and eventually leads to neuronal apoptosis and aggravated neural damage [20]. Studies have shown that, in chronic unpredictable mild stress (CUMS)-induced animal models of depression, the expression of ERS markers such as glucose-regulated protein 78 (GRP78), CCAAT/enhancer-binding protein homologous protein (CHOP), and X-box binding protein 1 (XBP1) is significantly upregulated in emotion-related brain regions including the hippocampus and prefrontal cortex, and is closely associated with depression-like behaviors [7]. Human studies have also shown that ERS markers such as GRP78 and CHOP are markedly elevated in the hippocampus of patients with MDD [21]. Therefore, aberrant activation of UPR-mediated apoptotic signalling may represent an important basis linking ER dysfunction with depression-related neuropathological changes.

Neuroinflammation is one of the important pathological mechanisms of depression. Studies have shown that ERS can target UPR-related signalling pathways, including PERK, IRE1α, and ATF6, and synergistically promote a pro-inflammatory microenvironment mediated by JNK and nuclear factor-κB (NF-κB). This upregulates pro-inflammatory cytokines and induces and amplifies neuroinflammation [22,23,24]. After ERS activation, the PERK pathway, ERS induces phosphorylation of serine 51 at the N terminus of eukaryotic initiation factor-2α (eIF-2α), thereby attenuating protein translation and synthesis and inhibiting IκB synthesis. Because IκB is a key protein that maintains NF-κB in an inactive state and has a shorter half-life than NF-κB, reduced translation increases the NF-κB/IκB ratio and releases more free NF-κB. Free NF-κB is subsequently translocated into the nucleus, upregulates pro-inflammatory cytokines such as tumor necrosis factor-α (TNF-α) and interleukin-1β (IL-1β), and induces persistent inflammatory responses [25,26]. Meanwhile, activation of the eIF2α-ATF4-CHOP axis can further amplify inflammatory and cellular injury signals [27]. The IRE1α pathway recruits TRAF2 to activate JNK and IκB kinase (IKK); activated JNK phosphorylates activator protein 1 (AP1), whereas IKK phosphorylates IκB and promotes its degradation, thereby activating NF-κB. NF-κB and AP1 then translocate to the nucleus to participate in the transcription of inflammation-related genes [25]. Activated ATF6 can also enhance NF-κB signaling and increase the expression of pro-inflammatory cytokines, thereby aggravating inflammation [13,28]. A large body of evidence shows that the levels of pro-inflammatory cytokines are significantly increased in the blood, cerebrospinal fluid, and emotion-related brain regions of patients with depression and animal models, and are closely associated with depression-like behaviors and neural dysfunction [29,30]. Therefore, the JNK/NF-κB inflammatory signaling axis mediated by the UPR is considered an important molecular mechanism linking ER dysfunction to the neuroinflammatory phenotype of depression. In addition, prolonged UPR signaling may lead to excessive ROS accumulation, further amplifying inflammatory and neuronal injury signals. During ERS, aberrant activation of protein disulfide isomerase (PDI), endoplasmic reticulum oxidoreductin-1 (ERO1), and the nicotinamide adenine dinucleotide phosphate (NADPH) oxidase complex leads to substantial ROS accumulation, in which the CHOP-ERO1α-IP3R1-CaMKII pathway plays a key role in ROS generation [23,31]. ROS can further activate MAPK and NF-κB signaling, forming a positive feedback loop of “ERS-oxidative stress-inflammation,” thereby aggravating neuronal injury and promoting the onset and progression of depression [13,32].

In summary, the PERK, IRE1α, and ATF6 branches of the UPR do not act in isolation; rather, they jointly participate in the onset and progression of depression by regulating apoptosis, inflammatory responses, and oxidative stress. This suggests that the UPR may serve as an important molecular hub linking ERS with depression-related neural injury.

### 3.2. Calcium Dyshomeostasis

ERS can cause ER calcium depletion or calcium overload, thereby interfering with protein synthesis, folding, and modification and ultimately triggering irreversible apoptosis [33]. Apoptosis is one of the important mechanisms underlying neuronal injury after the onset of depression [34]. Studies have shown that, in CUMS-induced rat models of depression, neuronal apoptosis in the hippocampus is closely associated with neuronal injury caused by elevated free Ca^2+^ concentrations in synaptosomes [35]. During ERS, inositol 1,4,5-trisphosphate receptors (IP3Rs) and ryanodine receptor (RyR) channels located on the ER membrane become activated, promoting substantial Ca^2+^ release from the ER lumen into the cytoplasm. This process subsequently activates calpains and induces cleavage of Caspase-12, thereby initiating the downstream caspase cascade and apoptosis. At the same time, the large amount of Ca^2+^ released from the ER into the cytoplasm can activate calmodulin-dependent proteases that act on substrates such as Bax or Bid and induce apoptosis. Moreover, under ERS conditions, stromal interaction molecule 1 (STIM1), a major component of store-operated calcium entry, translocates and interacts with ORAI1, a calcium release-activated calcium channel protein, thereby activating store-operated calcium channels, inducing massive Ca^2+^ influx, causing calcium overload, and ultimately triggering apoptosis [36,37]. Therefore, ERS-mediated disruption of Ca^2+^ homeostasis may jointly promote neuronal injury through ER calcium release, extracellular calcium influx, and activation of apoptotic signalling, thereby participating in the pathological process of depression.

ER-Ca^2+^ imbalance can also amplify cellular injury signals through ER-mitochondrial calcium transfer. Studies have shown that mitochondrial Ca^2+^ homeostasis dysregulation is associated with the pathophysiological mechanisms of depression. After large amounts of Ca^2+^ released from the ER enter the cytoplasm, they can be taken up by mitochondria, leading to mitochondrial Ca^2+^ overload, depolarization, opening of the mitochondrial permeability transition pore (MPTP), release of cytochrome c, and activation of caspases, ultimately inducing mitochondria-dependent apoptosis [38]. Excessive Ca^2+^ entry into mitochondria may also increase mitochondrial metabolic burden, promote ROS generation, maintain a highly permeable MPTP state, further activate caspase-9 and caspase-3, and aggravate neuronal apoptosis [39,40]. In addition, mitochondria are major sources of oxygen free radicals and therefore participate directly in oxidative stress, which is widely regarded as an important risk factor for depression. Extensive studies have shown that oxidative stress mediated by mitochondrial dysfunction is closely associated with depression [38]. During ERS, Ca^2+^ released from the ER and captured by adjacent mitochondria may further aggravate mitochondrial damage, increase free radical generation, and strengthen pro-apoptotic signaling. In addition, IRE1α has been reported to interact with Bak and Bax, thereby enhancing mitochondria-dependent cell death and contributing to depression development [21].

Collectively, these findings indicate that ERS can disrupt Ca^2+^ homeostasis through IP3R/RyR-mediated ER-Ca^2+^ release, STIM1-ORAI1-mediated store-operated calcium entry, and ER-mitochondrial calcium transfer. These processes induce mitochondrial dysfunction, oxidative stress, and caspase-dependent neuronal apoptosis, ultimately participating in depression-related neural injury.

### 3.3. Autophagy

Autophagy is an essential intracellular process responsible for maintaining neuronal homeostasis through the clearance of damaged organelles and aberrant protein aggregates. Its dysfunction is regarded as one of the key mechanisms underlying the onset and progression of depression. Under transient or mild ERS, the UPR may cooperate adaptively with autophagy by promoting the clearance of misfolded proteins, reducing the ER protein load, and restoring cellular homeostasis, thereby exerting protective effects. However, when ERS persists or becomes excessive, the compensatory capacity of the UPR gradually fails, ER homeostasis becomes difficult to restore, and autophagic flux is subsequently impaired. Abnormal proteins and damaged organelles continue to accumulate and further amplify ERS responses, forming a vicious cycle of “sustained ERS activation-autophagy impairment-aggravated cellular injury”. This ultimately induces apoptosis or necrosis and promotes neural injury and depression-like behaviours [41,42].

ERS-induced calcium dyshomeostasis may reduce autophagy and thereby contribute to depression [43]. Specifically, calcium dyshomeostasis induced by ERS is an important upstream factor in autophagic dysfunction. ERS disrupts the balance of Ca^2+^ storage and release within the ER, interferes with autophagosome-lysosome fusion, affects the activity and interactions of fusion-related proteins, and reduces autophagic degradation efficiency [44]. At the same time, abnormal Ca^2+^ signaling can hinder autophagosome and lysosome transport along microtubules toward fusion sites by affecting cytoskeletal dynamics [45]. Studies have also shown that excessively high cytosolic Ca^2+^ concentrations can inhibit autophagosome formation by activating IP3R-related pathways, thereby further weakening autophagic function [46].

Among the three major branches of the UPR, IRE1 signalling is closely linked to autophagy, inflammation, and cell death. In mammals, oligomerized IRE1 in mammalian cells not only cleaves the messenger RNA (mRNA) of X-box binding protein 1 (XBP1), but can also influence apoptosis by suppressing autophagy processes that interact with Caspase-12 and by activating stress-induced JNK. Inhibition of autophagy promotes the binding of IRE1 to TRAF2, stabilizes the conformation of IRE1, facilitates its interaction with ASK1, activates the IRE1-ASK1-JNK axis, and ultimately accelerates apoptosis [47]. Autophagy and apoptosis are both indispensable for the preservation of cellular homeostasis and normal tissue architecture. Dysregulation of either process may induce hippocampal neuronal injury while impairing neuroplasticity and regenerative capacity [48], thereby potentially inducing depression. Existing studies further support the link between reduced autophagy and depression, as chronic stress-induced depression models are often accompanied by decreased autophagy [49]. In hippocampal tissue of mice with postpartum depression, key autophagy-related proteins including Beclin1, ATG5, and LC3B have been reported to be significantly downregulated, accompanied by increased neuronal apoptosis, reduced neuronal density, and pathological alterations such as degeneration and necrosis. Clinically, decreased autophagic activity has likewise been observed in patients with postpartum depression [50]. Together, these findings suggest that sustained ERS may represent an important molecular mechanism of depression by inhibiting autophagic flux, amplifying calcium homeostasis disruption, and enhancing IRE1-JNK apoptotic signalling. Nevertheless, several key gaps remain. The specific molecular mechanisms by which ERS suppresses autophagy have not been fully clarified; the coordinated regulatory networks and temporal-intensity mechanisms linking autophagy with calcium dyshomeostasis and inflammation have yet to be fully clarified; the “double-edged sword” effect of autophagy in depression and the heterogeneity of autophagic regulation across different depression subtypes have not been explored; and current pharmacological tools targeting autophagy are highly non-selective, with insufficient evaluation of their long-term effects on normal neuronal homeostasis.

**Figure 1:**
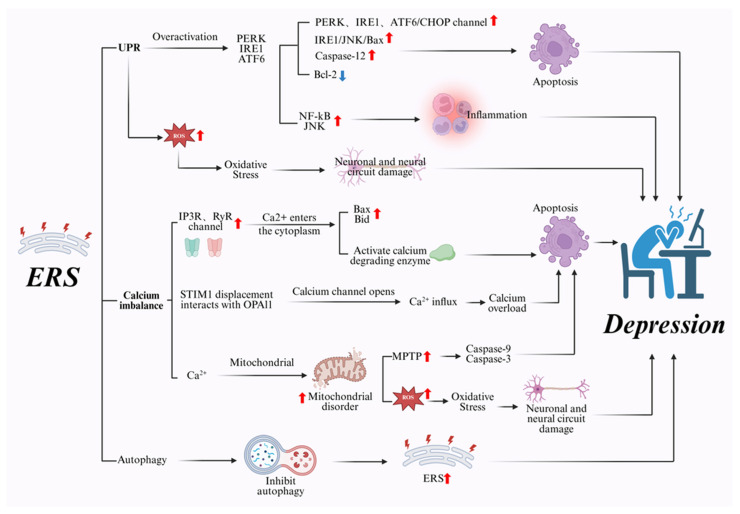
Role of endoplasmic reticulum stress in depression.

In summary, excessive activation of the UPR, disruption of Ca^2+^ homeostasis, and inhibition of autophagy under ERS can trigger apoptosis, inflammatory responses, and oxidative stress, thereby damaging neural cells and neurons and ultimately inducing depression (Figure 1).

## 4. Mechanisms by Which Endoplasmic Reticulum Stress Participates in Exercise-Induced Improvement of Depression

### 4.1. Exercise-Mediated Regulation of the Unfolded Protein Response

#### 4.1.1. Exercise- and UPR-Mediated Apoptosis

The unfolded protein response (UPR) plays a key role in the onset and progression of depression [4]. For example, human studies have shown that the expression levels of GRP78, GRP94, and calreticulin in the temporal cortex are higher in patients with MDD who died by suicide than in non-suicide death groups or other MDD groups [51]. Another study involving 86 patients with MDD also found that GRP78, EDEM1, CHOP, and XBP1 expression levels were significantly higher than those in control participants [52]. These changes may be associated with activation of the UPR-mediated PERK/CHOP signalling pathway, which further triggers apoptotic signalling, including downregulation of the anti-apoptotic protein Bcl-2 and upregulation of the pro-apoptotic proteins caspase-12 and caspase-3 [4]. Interestingly, 12 weeks of treadmill exercise (12 m/min, 60 min/day, 5 times/week) inhibited excessive activation of UPR-related pathways, including PERK/eIF2α, IRE1α/XBP1, and ATF6, in the brains of PS2 mutant mice and blocked Aβ-induced apoptotic signalling, thereby improving cognitive dysfunction [26]. This suggests that exercise may exert neuroprotective effects by regulating ERS and may represent a potential entry point for improving psychiatric disorders. The CUMS model is widely used to simulate depression-like behaviours [53,54]. One study showed that eight weeks of treadmill exercise performed for 21 days (19 m/min, 60 min/day, 5 times/week) activated the CREB/BDNF signalling pathway, inhibited the expression of ERS- and apoptosis-related proteins including GRP78, CHOP, and caspase-12, and improved CUMS-induced hippocampal ERS and memory impairment in mice [55]. Brain-derived neurotrophic factor (BDNF), a key factor regulating the development and function of neural circuits, has been considered an important biomarker of depression [56]. Treadmill exercise (18–20 m/min, 60 min/day, 6 times/week for 4 weeks) can also activate the BDNF/TrkB signalling pathway, inhibit the key ERS-related PERK/eIF2α/CHOP pathway, reduce apoptosis, and improve CUMS-induced depression-like behaviours in rats [57]. In addition, four weeks of treadmill exercise (20 m/min, 60 min/day, 6 times/week) increased hippocampal sigma-1 receptor (S1R) protein expression, thereby inhibiting apoptosis mediated by excessive activation of the GRP78/IRE1/XBP1/CHOP signalling pathway and improving CUMS-induced depression-like behaviours in rats [58]. Moreover, fluoride exposure can reduce neurotransmitter levels such as 5-HT and GABA and induce anxiety- and depression-like behaviours [59]. Voluntary wheel running improves sodium fluoride exposure-induced depression-like behaviours in mice through PERK/CHOP axis-mediated anti-apoptotic effects; however, all-day voluntary wheel running is more effective than exercise restricted to specific time periods such as morning or evening [15]. More importantly, Ding et al. found that prolonged swimming exercise characterized by exhaustion or fatigue-induced sinking can cause Ca^2+^ overload in isolated hippocampal cells of mice and further induce hippocampal synaptic plasticity damage through ERS-mediated apoptotic pathways [60]. Although this study did not assess changes in depression levels, it indicates that the potential risks of overtraining for brain health should be carefully considered.

In summary, exercise interventions may exert antidepressant effects by inhibiting excessive activation of ERS-mediated UPR pathways, maintaining the balance between pro- and anti-apoptotic proteins, and optimizing neurotransmitter systems. However, current research in this field remains exploratory, with evidence mainly derived from animal models and limited direct confirmation from human clinical studies. In addition, exercise effects are influenced by factors such as exercise mode (voluntary vs. forced), duration (moderate vs. exhaustive), and circadian rhythm, resulting in heterogeneity across protocols. Future studies should define optimal “exercise prescriptions” for depression and further explore the cross-species consistency of exercise-mediated regulation of protein homeostasis, thereby providing a more scientific theoretical basis for precise prevention and treatment of depression.

#### 4.1.2. Exercise- and UPR-Mediated Inflammation

**Figure 2:**
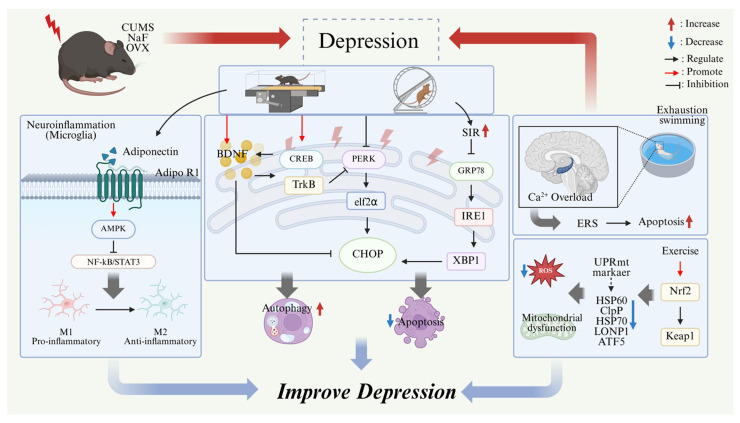
Mechanisms by Which Endoplasmic Reticulum Stress Participates in Exercise-Induced Improvement of Depression.

In recent years, accumulating evidence has highlighted the critical role of neuroinflammation in the onset and progression of depression. Clinical studies have demonstrated that patients with major depressive disorder (MDD) exhibit significantly elevated levels of inflammatory markers in the peripheral circulation compared with healthy individuals [61]. During this process, endoplasmic reticulum stress (ERS)-mediated unfolded protein response (UPR) plays a pivotal role in depression-related inflammatory responses. Among the molecular regulatory branches of ERS, the PERK-eIF2α, IRE1α, and ATF6α pathways function as key upstream mechanisms driving inflammatory signaling activation, while persistent ERS exacerbates inflammation and promotes the development of depression [62]. Following exercise intervention, disturbances in ER homeostasis are effectively alleviated, accompanied by suppressed expression of ERS-related proteins such as PERK, IRE1α, and ATF6α, as well as reduced activation of downstream stress signaling pathways. These findings suggest that exercise may attenuate inflammatory responses through restoration of ER homeostasis [63]. A previous study involving three months of treadmill exercise in mice demonstrated that treadmill training downregulated GRP78 expression and inhibited the activation of PERK, eIF2α, and ATF4 [64]. Suppression of the PERK/eIF2α/ATF4 pathway can effectively alleviate oxidative stress and inflammation, thereby exerting neuroprotective and antidepressant effects [65,66]. Meanwhile, animal studies have revealed that mice exposed to chronic social defeat stress (CSDS) not only exhibited reduced dendritic spine density and decreased PSD95 protein levels in the hippocampus, but also showed activation of ERS-related signaling pathways (PERK, p-eIF2α, CHOP, and XBP1), polarization of microglia toward the pro-inflammatory M1 phenotype, and significantly elevated expression of inflammatory cytokines such as IL-1β and TNF-α [67]. Further investigations demonstrated that targeting PERK-mediated ERS effectively ameliorated lipopolysaccharide (LPS)-induced pro-inflammatory responses in hippocampal microglia and depressive-like behaviors in mice [68]. Therefore, suppressing ERS and blocking the pro-inflammatory polarization of microglia may represent promising therapeutic targets for depression. Current evidence further indicates that adiponectin, a hormone primarily secreted by adipose tissue, participates in multiple biological processes, including neuroprotection and anti-inflammatory regulation [69]. Four weeks of moderate-intensity treadmill exercise were shown to maintain the balance of microglial M1/M2 polarization through activation of the hippocampal adiponectin/AdipoR1-mediated AMPK-NF-κB/STAT3 signaling pathway, thereby alleviating CUMS-induced neuroinflammation and depressive-like behaviors in mice [70]. Notably, although these studies confirmed that exercise-induced adiponectin signaling exerts antidepressant effects through modulation of microglial polarization, the upstream molecular targets involved have not yet been fully elucidated. Future studies are needed to further clarify the interaction network between adiponectin signaling and hippocampal ERS, which may provide deeper theoretical insights into the antidepressant mechanisms of exercise. (Figure 2, Table 1).

### 4.2. Exercise-Mediated Regulation of Calcium Homeostasis

ER-Ca^2+^ imbalance can amplify cellular injury through excessive ER-mitochondrial calcium transfer, leading to mitochondrial dysfunction and thereby contributing to the pathophysiology of depression [38]. Mitochondria-associated ER membranes (MAMs) are an important component of intracellular signal transduction. Under physiological conditions, Ca^2+^ released from the ER is transported to mitochondria through MAMs, thereby maintaining normal mitochondrial metabolism [71]. A large number of studies have shown that exercise produces broad benefits through MAM-mediated pathways in the heart [72], skeletal muscle [73], liver [74], and whole-body metabolic homeostasis [75]. However, little is known about whether exercise produces psychological benefits by regulating ER-mitochondrial communication. A recent study showed that six weeks of treadmill exercise (10 m/min, 75 min/day, 5 times/week) activated the classical antioxidant Nrf2/Keap1 signalling pathway, suppressed the mRNA levels of mitochondrial UPR (UPRmt) markers including HSP60, ClpP, HSP70, LONP1, and ATF5, as well as the ERS transcription factor CHOP, restored mitochondrial function, and improved CUMS-induced hippocampal oxidative damage and depression-like behaviours such as anxiety, anhedonia, and behavioural despair [12]. This finding opens a new perspective for elucidating the molecular mechanisms underlying the antidepressant effects of exercise. However, future research should comprehensively explore this issue at cellular, animal, and human levels to systematically clarify how exercise interventions reshape the structure and function of neuronal MAMs in depression models. (Figure 2, Table 1).

### 4.3. Exercise-Mediated Regulation of ERS-Related Autophagy

Autophagy and apoptosis are two key processes that determine cell fate. ERS-induced UPR can trigger protective autophagy to counteract apoptosis [76]. Previous studies have shown that high-fat diet-induced insulin-resistant mice exhibit significantly increased expression of ERS-related proteins, including IRE1, PERK, and ATF6, and significantly decreased levels of autophagy markers including BNIP3, LC3II/LC3I, and PINK1. Eight weeks of swimming exercise activated the AMPK/PGC-1α signalling pathway, inhibited insulin resistance-induced ERS, and promoted protective autophagy [77]. This suggests that exercise may restore cellular homeostasis and exert potential health benefits by regulating the ERS-autophagy axis. Although relatively few studies have examined the antidepressant effects of exercise by targeting this pathway, one study found that four weeks of uphill running (15–20 m/min, 5° incline, 60 min/day, 5 times/week) promoted autophagy by increasing LC3-II and Beclin-1 protein expression, inhibited activation of the ERS-related PERK/eIF2α/CHOP signalling pathway, reduced caspase-3 protein expression and apoptosis, and thereby improved learning and memory impairment as well as depression-like behaviours induced by chronic stress after ovariectomy in rats [42]. These results indicate that exercise not only improves autophagic function but also suppresses neuronal apoptosis through autophagy-mediated ERS remodelling, thereby achieving antidepressant neuroprotection. However, whether other forms of exercise, such as resistance training and high-intensity interval training, have beneficial effects on autophagy-related ERS and subsequent depression-like behaviours remains unreported. Thus, further exploration is needed. (Figure 2, Table 1).

## 5. Conclusions

This review provides a comprehensive summary of recent progress regarding the role of exercise in counteracting ERS-mediated pathological injury and improving depressive symptoms. Increasing evidence indicates that exercise interventions alleviate depression-like behaviours through ERS-mediated multi-pathway integration, including maintenance of ER and mitochondrial functional homeostasis, inhibition of neuronal apoptosis, attenuation of neuroinflammation, and enhancement of autophagic activity. However, current research on the role of ERS in exercise-induced improvement of depression is mainly based on animal studies and lacks clinical experimental evidence. One of the most challenging barriers is the development of personalized exercise programmes for patients. Therefore, the type and duration of exercise require further investigation through well-designed clinical trials and broader application. Future studies should also explore combined strategies involving exercise and pharmacological interventions, such as ERS inhibitors represented by ISRIB-like compounds, to enhance antidepressant efficacy through additive effects. At the same time, the molecular mechanisms by which exercise targets ERS to exert antidepressant effects remain insufficiently understood. Future studies should integrate multi-omics technologies, such as phosphoproteomics and epigenomics, with gene-editing approaches, such as CRISPR-Cas9-mediated knockout of key ERS genes, to systematically elucidate how exercise coordinates cellular homeostasis and the balance between inflammation and apoptosis through ERS, and to identify potential targets such as spliced XBP1 and GRP78/BiP. In addition, current studies rely largely on traditional molecular biology techniques, which makes it difficult to dynamically track the spatiotemporal characteristics of ERS. Future research may introduce live-cell imaging, such as fluorescent reporter systems for monitoring ER calcium oscillations, spatial transcriptomics to resolve brain region-specific ERS patterns, single-cell sequencing to identify vulnerable neuronal subpopulations, and metabolic flux analysis, such as 13C-labelled tracing of ER-mitochondrial metabolite exchange. These approaches may reveal the dynamic network through which exercise modulates ERS from multiple dimensions.

## Figures and Tables

**Table 1:** Research summary of exercise intervention regulating ERS and related signaling pathways.

Reference	Model	Exercise Modality	Exercise Protocol	Main Effects
[55]	Prolonged restraint	8 weeks of treadmill running	19 m/min, 60 min/day, 5 times/week	↓GRP78, CHOP, Cleaved caspase-12 CREB/BDNF pathway activation
[57]	CUMS	4 weeks of treadmill exercise	18–20 m/min, 60 min/day, 6 times/week for 4 weeks	ERS protein(↓PERK, eIF2α, CHOP), anti-apoptotic(↑Bcl-2, BDNF and TrkB), Activation of BDNF/TrkB signaling pathway, Inhibition of PERK/elf2α/CHOP pathway.
[58]	CUMS	treadmill exercise	20 m/min, 60 min/day, 6 times/week	↑Hippocampus SIR protein ↓GRP78, IRE1, XBP1, CHOP, caspase-3 Inhibition of GRP78/IRE1/XBP1/CHOP signaling pathway
[15]	Sodium fluoride	Voluntary Wheel Running	daytime/night/all day	PERK-CHOP pathway ↓eIF2α, ATF4, Caspase-12, Caspase-3, CHOP, Bcl-2, ATF6, p-eIF2α neuroplasticity-related proteins (↑BDNF, PSD95, NMDAR1, SYN)
[60]	Intense exercise	swimming	Exhaustion	↑GFAP, Bax, caspase-3, caspase-12, CHOP, p-JNK ↓SYP, synapse plasticity, Bcl-2
[70]	CUMS	running exercise	Running exercise for 4 weeks	↓microglial numbers, ↑adiponectin levels in adipose tissue, muscle and plasma, activation of AMPK-NF-κB/STAT3 signaling pathway, inhibition of hippocampal neuroinflammation ↓depressive-like behaviors,
[12]	CUMS	6-week treadmill exercise	10 m/min, 75 min/day, 5 times/week	restored mitochondrial functions (↑Δψm, ATP, ↓ROS) eliminated oxidative stress (↑SOD, T-AOC, ↓MDA) UPRmt markers (↓HSP60, ClpP, HSP70, LONP1, and ATF5), ↓CHOP Activation of the Nrf2/Keap1 pathway (↑Nrf2, NQO1, HO-1; ↓Keap1) ↓depression-like behaviors
[42]	Ovx and CUMS	4-week treadmill exercise	15–20 m/min, 5° incline, 60 min/day, 5 times/week	ERS-related proteins (↓PERK, eIF2α, CHOP) ↓Caspase-3, Autophagy markers (↑Beclin-1, LC3-II)

Table note: ERS, endoplasmic reticulum stress; GRP78, glucose-regulated protein 78; CHOP, C/EBP homologous protein; BDNF, brain-derived neurotrophic factor; CREB, cAMP response element-binding protein; PERK, protein kinase R-like endoplasmic reticulum kinase; eIF2α, eukaryotic translation initiation factor 2α; TrkB, tyrosine kinase receptor B; IRE1, inositol-requiring enzyme 1; XBP1, X-box binding protein 1; ATF4/ATF6, activating transcription factor 4/6; Bcl-2, B-cell lymphoma 2; Bax, Bcl-2-associated X protein; caspase, cysteinyl aspartate-specific proteinase; GFAP, glial fibrillary acidic protein; p-JNK, phosphorylated c-Jun N-terminal kinase; SYP, synaptophysin; AMPK, AMP-activated protein kinase; NF-κB, nuclear factor kappa-B; STAT3, signal transducer and activator of transcription 3; UPRmt, mitochondrial unfolded protein response; HSP60/HSP70, heat shock protein 60/70; LONP1, lon peptidase 1, mitochondrial; Nrf2, nuclear factor erythroid 2-related factor 2; Keap1, Kelch-like ECH-associated protein 1; NQO1, NAD(P)H: quinone oxidoreductase 1; HO-1, heme oxygenase 1; LC3, microtubule-associated protein 1 light chain 3; Beclin-1, autophagy-related protein; Δψm, mitochondrial membrane potential; ROS, reactive oxygen species; SOD, superoxide dismutase; T-AOC, total antioxidant capacity; MDA, malondialdehyde. ↑ Increase, activation, or upregulation; ↓ Decrease, inhibition, or downregulation.

## Data Availability

The original contributions presented in this study are included in the article. Further inquiries can be directed to the corresponding author.
